# Nurses’ Use of Personal Smartphone Technology in the Workplace: Scoping Review

**DOI:** 10.2196/18774

**Published:** 2020-11-26

**Authors:** Andrea de Jong, Lorie Donelle, Michael Kerr

**Affiliations:** 1 Arthur Labatt Family School of Nursing Faculty of Health Sciences Western University London, ON Canada

**Keywords:** nurses, digital health, smartphone, evidence-informed practice

## Abstract

**Background:**

There has been an increase in the technological infrastructures of many health care organizations to support the practice of health care providers. However, many nurses are using their personal digital devices, such as smartphones, while at work for personal and professional purposes. Despite the proliferation of smartphone use in the health care setting, there is limited research on the clinical use of these devices by nurses. It is unclear as to what extent and for what reasons nurses are using their personal smartphones to support their practice.

**Objective:**

This review aimed to understand the current breadth of research on nurses’ personal smartphone use in the workplace and to identify implications for research, practice, and education.

**Methods:**

A scoping review using Arksey and O’Malley’s methodological framework was conducted, and the following databases were used in the literature search: CINAHL, PubMed, ProQuest Dissertations and Theses, Embase, MEDLINE, Nursing and Allied Health Database, Scopus, Web of Science, and Cochrane Reviews. Search terms used were Nurs* AND (personal digital technology OR smartphone OR cellphone OR mobile phone OR cellular phone). Inclusion criteria included research focused on nurses’ use of their own digital technologies, reported in English, and published between January 2010 and January 2020. Exclusion criteria were if the device or app was implemented for research purposes, if it was provided by the organization, if it focused on infection control, and if it was focused on nursing students or nursing education.

**Results:**

A total of 22 out of 2606 articles met the inclusion criteria. Two main themes from the thematic analyses included *personal smartphone use for patient care* and *implications of personal smartphone use*. Nurses used their smartphones to locate information about medications, procedures, diagnoses, and laboratory tests. Downloaded apps were used by nurses to locate patient care–related information. Nurses reported improved communication among health team members and used their personal devices to communicate patient information via text messaging, calling, and picture and video functions. Nurses expressed insight into personal smartphone use and challenges related to distraction, information privacy, organizational policies, and patient perception.

**Conclusions:**

Nurses view personal smartphones as an efficient method to gather patient care information and to communicate with the health care team. This review highlights knowledge gaps regarding nurses’ personal device use and information safety, patient care outcomes, and communication practices. This scoping review facilitates critical reflection on patient care practices within the digital context. We infer that nurses’ use of their personal devices to communicate among the health care team may demonstrate a technological “work-around” meant to reconcile health system demands for cost-efficiency with efforts to provide quality patient care. The current breadth of research is focused on acute care, with little research focus in other practices settings. Research initiatives are needed to explore personal device use across the continuum of health care settings.

## Introduction

Smartphone use is increasing globally, with an estimated 3.3 billion users in 2019 and an anticipated 3.8 billion users by 2021 [1]. Countries with the greatest number of smartphone users include China, India, and the United States [1]. A smartphone is a device that has both computing abilities and mobile communication technology [2]. Mobile communication technology has undergone rapid development [3], and smartphone production is one of the fastest growing technological industries [4]. Coupled with the proliferation of smartphones is the high demand for downloadable apps that a smartphone user can add to their device. In 2018, over 4 million Android and Apple apps were available either free or for a cost [5]. These apps provide the user with additional services and features on their smartphone device.

Within the health care context, there has been an increase in the use of smartphones by health care providers in the last several years [2], including an increase in the use of health professional apps [6]. Fölster [7] reported over 200,000 health-related apps available for purchase or free of charge. The increased use of smartphones and selected apps by health care professionals mirrors the increased use of information technology within health care. There has been a significant increase in the implementation and use of electronic documentation systems, with billions of dollars being utilized to support the provision and coordination of health care to patients using information technology [8]. However, despite the increased use of apps in health care, health care professionals have expressed concerns relating to the trustworthiness of apps and a knowledge gap on the effectiveness of apps [9].

Electronic documentation systems have been integrated within health care systems to provide a centralized repository and accessible source of clinical information for health care providers [10]. These same systems are intended to support clinical care practices among all health care providers while providing access to information, decision-support tools, and improved workflow [10,11]. Given their extended contact with patients and families, nurses tend to have the greatest interaction with electronic documentation systems relative to other health care providers [12].

Despite the increase in technological infrastructures of many health care organizations to support the practice of health care providers, many nurses are using their personal digital devices (eg, smartphones) while at work for both professional and personal purposes [13,14]. Yet, there is limited research available on the clinical use of personal smartphone devices by nurses. In addition, there is limited research available that focuses on nurses’ use of commercially available health-related apps in the workplace [15]. These gaps in research are especially important to understand given the most recent global pandemic caused by COVID-19 [16]. The COVID-19 pandemic lead to rapidly evolving public health measures with frequent changes in practice guidelines in all health care settings. Health care organizations had to respond and adapt quickly. With smartphones being a convenient and accessible way to locate information, the use of these devices in practice by nurses needs to be an area of focus, especially when facing a global pandemic.

It is unclear as to what extent and for what reasons nurses are using their personal smartphones to support their practice. Similarly, there is a lack of clarity regarding the quality and credibility of the resources accessed by nurses via their personal devices. However, the expectation of evidence-informed patient care practices highlights the importance of access to health-related resources for nurses and other health care providers [17]. In essence, evidence-informed practice necessitates nurses to critically evaluate information collected regarding patients’ needs and to integrate it with the available clinical and research evidence to enable evidence-informed patient care [18]. A valued feature of mobile smartphones is the *pro re nata,* or PRN, access to vast amounts of online health-related information. In this scoping review, we are interested in how nurses are leveraging their personal smartphones for personal and patient care–related purposes across a variety of health care settings.

This research aims to explore and synthesize the current literature regarding nurses’ use of their personal digital devices in the workplace. The purpose of this review is to understand the current breadth of research to identify knowledge gaps, practice and policy implications, and future research opportunities. More specifically, this review seeks to gain an understanding of nurses’ use of their personal digital technology within the workplace.

## Methods

### Overview

A scoping review was the chosen method to review the literature as it is well suited for burgeoning areas of research. In addition, scoping reviews aim to identify gaps in research while mapping key concepts and types of evidence [19]. Arksey and O’Malley’s [20] methodological framework outlines five stages for conducting a scoping review, including identifying the research question; identifying relevant studies; study selection; charting the data; and collating, summarizing, and reporting the results. A critical step in this final stage involves identifying the research and evidence gaps that need to be addressed [20]. The PRISMA-ScR (Preferred Reporting Items for Systematic Reviews and Meta-Analyses extension for Scoping Reviews) checklist was used to ensure that the scoping review is robust and includes all essential reporting items [21].

### Step 1: Identify the Research Question

The research question we identified for this review is “How are nurses using their personal smartphones within the clinical workplace?”

### Step 2: Identifying Relevant Studies

The following databases were used in the literature search process: CINAHL, PubMed, ProQuest Dissertations and Theses, Embase, MEDLINE, Nursing and Allied Health Database, Scopus, Web of Science, and Cochrane Reviews. Search terms used were: Nurs* AND (personal digital technology OR smartphone OR cellphone OR mobile phone OR cellular phone). Different search terms were trialed during the initial literature search process. The search terms selected were broad and were determined to capture all relevant studies that relate to the research question. See Multimedia Appendix 1 for the full syntax of search terms.

Articles included in the review had to meet the following inclusion criteria: a research focus on nurses’ use of their own digital technology, research reported in English, and research published between January 2010 and January 2020 in order to capture data that aligned with the proliferation of mobile phone ownership. Studies focusing on a specific device or online app use were included only if the motivation for use was nurse driven. Studies were excluded from this review if the workplace organization provided the technology or if the technology was implemented for research purposes. Studies that focused on infection control of personal digital technology were excluded. Studies were also excluded if they focused on personal digital technology use and nursing students or nursing education, as this review was aimed at understanding practicing nurses’ use of digital health technologies within clinical settings.

### Step 3: Study Selection

**Figure 1:**
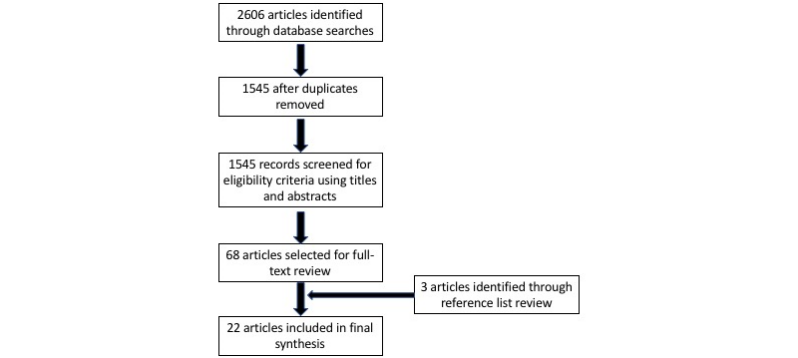
Flow diagram for article selection process.

A total of 2606 studies were identified using the search criteria above. Through consensus agreement between two reviewers, 22 studies met the inclusion and exclusion criteria and were included for the scoping review. See Figure 1 for a flow diagram of the article selection process.

### Step 4: Charting the Data

Two databases were set up in Step 4. One database, Mendeley, was used for sharing of articles between authors, including sharing to achieve consensus review. The other database was a matrix where all the data were collected. A spreadsheet was created to organize relevant information from the included articles. Data collected from each article included author names, year of publication, study design, data collection method, target population, study location, and research question.

### Step 5: Collating, Summarizing, and Reporting the Results

During the final stage of collating, summarizing, and reporting the results, the framework approach described by Ritchie and Spencer [22] and used by Arksey and O’Malley [20] was used in this review. This technique involves synthesizing and interpreting the data by sifting, sorting, and charting the information based on the key themes and issues presented in the literature [22]. The data were charted and thematically analyzed. Data analysis was conducted by two researchers and analytical discrepancies were resolved through discussion until consensus was achieved.

## Results

### Overview

The 22 studies included in this research (see Tables 1-3 [23-44]) were conducted across a diverse range of countries and published between 2013 and 2019, with most (17/22, 77%) published in 2016 or later. Out of the 22 included studies, 16 (73%) used quantitative (ie, cross-sectional survey) research designs [23-38], 4 (18%) were qualitative [39-42], and 2 (9%) used a mixed methods design [43,44].

**Table 1:** Included quantitative studies.

Study design and author (year)		Data collection method		Target population		Study location		Research questions		Negative implications of personal digital technology		Purpose for digital technology use
Alameddine et al (2019) [23]	Survey, cross-sectional		97 emergency department (ED) providers, including ED faculty members, attending physicians, medical students, residents, and nurses (33% nurses)		Academic health center with the highest volume of patient visits in Lebanon		1. What are the frequency and patterns of smart device use among health care providers in the ED of a large academic health center in Lebanon?		Distraction Patient perception		Information seeking for clinical use Communication for clinical purposes Personal communication purposes Other personal uses	
Bautista (2019) [24]	Pen and paper survey		517 staff nurses		19 tertiary-level general hospitals in Metro Manila, Philippines		1. Using a ranking system, how do Filipino nurses use their smartphones for work purposes? 2. What are the differences in nurses' smartphone use for work purposes based on demographic and organizational factors?		N/A^a^		Communication for clinical purposes Information seeking for clinical use	
De Benedictis et al (2019) [25]	Survey		125 nurses and 66 physicians		An Italian university hospital in Rome, Italy		1. In what way is WhatsApp used in hospital settings by physicians and nurses with patients and between colleagues? 2. Which are the main perceived benefits and threats concerning the use of WhatsApp in a hospital setting by physicians and nurses? 3. Which are the determinants (individual and/or organizational) of the use of WhatsApp in a hospital setting? 4. Is there an interplay between individual and organizational determinants?		Perceived risks (privacy and confidentiality) Lack of organization regulations Distraction		Communication for clinical purposes Personal communication purposes	
Di Muzio et al (2019) [26]	Survey		193 nurses		Sapienza University Hospital, Rome, Italy		1. What is the validity and reliability of the Nurses' Use of PCDs^b^ Questionnaire in the Italian hospitals?		Increase error Negative impact on performance Distraction		Personal communication purposes Information seeking for clinical use Other personal uses	
Flynn et al (2018) [27]	Survey		735 acute care nurses, point of care and not point of care		Six acute care medical-surgical facilities in an urban health care system in the Southern United States		1. What are the current rates of personal smartphone use by nurses in acute care settings? 2a. What are nurses' preferences regarding the use of smartphone functionality in acute care settings? 2b. Are there differences in use by age category or role? 3a. What are nurses' perceptions of the benefits and drawbacks of using smartphones in the acute care setting? 3b. Are there differences in perceptions by age category or role?		May upset families Distraction Increase error		Information seeking for clinical use Communication for clinical purposes Personal communication purposes Other personal uses	
Garner et al (2017) [28]	Survey		97 acute care nurses and physicians (82.5% were nurses)		340+-bed tertiary facility in Bengaluru, India		1. What are the smartphone access and use, including future opportunities for mHealth^c^ and potential ethical implications, among health care professionals practicing at a health care facility in Bengaluru, India?		Confidentiality Misuse of health information Patient anxiety Cybercrime Limited Wi-Fi access		Communication for clinical purposes Personal communication purposes Other personal uses	
Grabowsky (2015) [29]	Survey		59 advanced practice nurses (APNs)		Alabama, United States; physician’s office, outpatient clinics, hospitals, academic health centers, employee health clinic, hospice, nurse practitioner–owned practice, Veterans Administration nursing home, urgent care, health department, and dialysis unit		1. What types of clinical questions are answered using smartphones? 2. Are there barriers to information seeking with smartphones? 3. What phone apps and online resources do APNs find most useful in clinical situations? 4. How do APNs view their current online searching skills? 5. What is the level of interest in receiving training in online searching and what type of training is preferred? 6. Is the use of smartphones to answer clinical questions related to gender, level of education, population of practice area, practice type, or years approved to practice as an APN?		Lack of internet access		Information seeking for clinical use	
Hranchook et al (2018) [30]	Survey		258 certified registered nurse anesthetists (CRNAs)		Michigan Association of Nurse Anesthetists, United States		1. What are the clinical and nonclinical uses of mobile computing devices among Michigan CRNAs? 2. What are the experiences of Michigan CRNAs with regard to the impact of using these devices on patient care?		Distraction Risk to patient Performance decline Policy		Information seeking for clinical use Communication for clinical purposes Personal communication purposes Other personal uses	
Mayer et al (2019) [31]	Survey		1293 nurses across a range of settings, including hospital care, primary care, social health care, prehospital care, management, teaching and research, and private practice		Nursing Association of Barcelona		1. Are nurses using health apps professionally and what types of apps are they using? 2. Among nurses, is there a need for training in the use of health apps? 3. What are nurses’ perceptions of health professional apps? 4. Is there a need for a certification process for health apps and what type of institution or organization should review and validate these apps for professional use?		Concern about information quality in health apps		Information seeking for clinical use	
McBride et al (2015) [32]	Survey		825 acute care hospital registered nurses (RNs)		Members of Academy of Medical Surgical Nurses, United States		1. What is the frequency of nonwork-related use of personal mobile phones and other personal communication devices among hospital RNs?		N/A		Personal communication purposes Other personal uses	
McBride and LeVasseur (2017) [33]	Survey		1268 nurses (staff, charge, advanced practice, managers, faculty, and executive)		Members of the Academy of Medical Surgical Nurses and the Society of Pediatric Nurses, United States		1. How do RNs working on inpatient units use their PCDs at work (excluding lunch and breaks) and what are their opinions about how PCD use impacted their work and the work of their colleagues?		Distraction Negative impact on performance		Information seeking for clinical use Communication for clinical purposes Personal communication purposes Other personal uses	
Mobasheri et al (2015) [34]	Survey		564 acute care nurses and 287 doctors		Five individual hospital sites in London, United Kingdom		1. What are the ways that frontline staff are using smartphones, tablet devices, and mHealth apps in the clinical environment?		Privacy		Communication for clinical purposes	
Moore and Jayewaedene (2014) [35]	Survey		82 acute care nurses and 334 doctors		40+ acute trusts in England		1. How do nurses and doctors use their smartphones at work, what do they use them for, and do they assess the risks associated with the apps they use?		Uncomfortable using phone in front of patients		Information seeking for clinical use	
Piscotty et al (2016) [36]	Survey		140 nurses in Registered Nurse to Bachelor of Science in Nursing program (RNs already had worked as nurses)		Public school of nursing in Southeast Michigan, United States		1. What is the prevalence of social media use by nurses during work hours?		N/A		Personal communication purposes Other personal uses	
Pucciarelli et al (2019) [37]	Survey		256 acute care nurses who worked in hospitals, outpatient facilities, or day surgeries		Seven hospitals in Central and Southern Italy		1. What are the work- and nonwork-related activities performed by nurses using smartphones in the workplace? 2. What are the differences between smartphone use and nurses' age, gender, and working environment? 3.What are the positive or negative influences that smartphones have on nurses' performance during their health care activities?		Distraction Negative impact on performance Inappropriate recording Inappropriate vital signs measuring		Information seeking for clinical use Communication for clinical purposes Personal communication purposes Other personal uses	
Stergiannis et al (2017) [38]	Survey		974 acute care medical and nursing staff (18.5% nursing assistants and 42.6% nurses, with the rest being doctors and junior doctors)		Six general hospitals in Athens, Greece		1. What is the clinical use of smartphones among medical and nursing staff in Greece?		Unaware of apps that can be used to assist them in their daily clinical task Internet access Did not think smartphones were useful Unsure about appropriate sites and apps Distrustful of information Lack of education on how to use phone		N/A	

^a^N/A: not applicable; this information was not reported in the study.

^b^PCD: personal communication device.

^c^mHealth: mobile health.

**Table 2:** Included qualitative studies.

Study design and author (year)		Data collection method		Target population		Study location		Research questions		Negative implications of personal digital technology		Purpose for digital technology use
Bautista and Lin (2016) [40]	Semistructured interview		30 acute care staff, charge, and nurse managers		13 tertiary hospitals in the Philippines		1. How do the interactions of sociotechnical components (users, technology, and policy) affect staff nurses’ use of personal mobile phones at work?		Personal cost, as hospital does not provide phone Distraction Privacy gaps and punishments Policy Patient complaints Infection control		Information seeking for clinical use Communication for clinical purposes	
Bautista and Lin (2017) [39]	Semistructured interview		20 acute care staff nurses		Nine hospitals in the Philippines		1. How and why are mobile instant messaging apps used by Filipino nurses as part of their work? 2. What are the gratifications derived by nurses when using mobile instant messaging apps?		N/A^a^		Communication for clinical purposes Personal communication purposes	
Chiang and Wang (2016) [41]	Semistructured interview		17 community nurses working for home care facilities		Two regional hospital-affiliated home care facilities and four community home care facilities in Southern Taiwan		1. What are nurses’ experiences regarding the benefits and obstacles of using a smart mobile device app in home care?		Perceived risks (privacy and confidentiality) Lack of organization regulations and incentives Disturbance to personal life, as messages received during nonworking hours		Communication for clinical purposes	
Park and Lee (2019) [42]	Semistructured focus group interviews		4 orthopedic scrub nurses		Operating room of a veterans’ hospital, Korea		1. How are scrub nurses using a commercially available smartphone app to solve information needs in orthopedic surgery?		N/A		Using an app to seek information for clinical use	

^a^N/A: not applicable; this information was not reported in the study.

**Table 3:** Included mixed methods studies.

Study design and author (year)		Data collection method		Target population		Study location		Research questions		Negative implications of personal digital technology		Purpose for digital technology use
Giles-Smith et al (2017)^a^ [43]	Survey, focus groups		94 acute care inpatient medical and surgical nurses		Community hospital and tertiary hospital in Winnipeg, Canada		1. What is the current usage of mobile devices and apps by nurses for direct patient care within the study sites? 2. What are the attitudes of nurses at these study sites toward the use of mobile devices and apps for direct patient care?		Lack of wireless internet Distraction Unsure if allowed to use at work Potential damage or loss of phone Infection control Concern of patient perception of phone Professionalism		Information seeking for clinical use	
Planitz et al (2013) [44]	Survey, observation		299 acute care nurses		Various units and wards in a tertiary hospital located in Brisbane, Australia		1. What is the level of actual personal smartphone use by nurses that is occurring within the hospital? 2. What are the attitudes of nurses toward smartphone use at the hospital? 3. Do the benefits of smartphones outweigh the consequences of distractions and occasional misuse? 4. What are the factors influencing whether nurses used smartphones at work? 5. How are nurses using their smartphones at work?		Disruptive		Personal communication purposes Other personal uses	

^a^Two-part study: only part 1 relates to the research question.

### Health Care Settings

In reviewing the included studies, most researchers focused on investigating personal smartphone use among hospital-based nurses (18/22, 82%) [23-28,30,32-35,37-40,42-44]. As well, 6 studies out of 22 (27%) included a multidisciplinary group that also included inquiry into physicians’ use of personal smartphones in the clinical setting [23,25,28,34,35,38]. There was a single study that investigated registered nurses’ personal technology use during work hours, whose sample population were enrolled in a Registered Nurse to Bachelor of Science in Nursing program, with 93% of the nurse participants currently working in acute care [36].

Out of 22 studies, 2 (9%) targeted nurses working in community-based health care settings [29,41]. Grabowsky [29] focused on advanced practice nurses who worked in an array of settings, including a variety of outpatient clinics. Chiang and Wang [41] investigated personal digital technology use among community nurses who worked for home care organizations. Out of 22 studies, 1 (5%) included nurses from a range of settings, both acute and nonacute, including hospital, primary care, social health care, prehospital care, management, education, and private practice settings [31].

Two main themes with associated subthemes were generated from the analyses. The first theme was *personal smartphone use for patient care*. This theme was divided into three subthemes: information seeking, communication, and mobile device functions. The second theme was *implications of personal smartphone use*, which was refined into five subthemes: smartphone use for personal reasons, distraction, patient perception, privacy and confidentiality, and organizational support and policy confusion. The details of the themes and subthemes are described narratively in the following sections.

### Personal Smartphone Use for Patient Care

#### Information Seeking

Nurses used their smartphones to access information directed at patient care. The most common information sought was related to medications, including drug guides and drug references, in 14 of the 22 (64%) studies reviewed [23,26-31,33-35,37,38,40,43]. Nurses reported efficiency of access to medication information; it was easier to search medications by either the generic name or brand name using their smartphones, despite having access to a drug reference book [40]. By using a mobile device, nurses were able to quickly look up new medications in drug guides [43]. Out of 22 studies, 8 (36%) identified that nurses used apps downloaded to their devices to find information on medications; 4 (18%) did not report the name of the apps used [23,27,34,35]. Whether nurses located medication information though a general search engine app (eg, Google) or by a specific app was not disclosed. The other 4 (18%) studies identified the specific apps used by nurses as Epocrates, Micromedex, Drug Index and Dosage, and Vademecum International [28,29,31,43].

Nurses also used their smartphones to access the internet for disease-related information [27,29,30,34,40,43]. Smartphones were used to collect procedural information, including various surgical procedures and anesthetic procedures [27,30,34,40,42]. Other than Park and Lee [42], researchers did not specify if nurses located this information specifically through online search engines or by specific apps they downloaded to their smartphone devices.

Nurses identified the need for information access regarding procedures and use of instruments within surgical settings [42]. The greatest barrier to information access included the inconvenience created by a lack of dedicated computers within surgical suites, thereby limiting nurses’ ability to access information [42]. Surgical nurses used the app, BAND, on their personal devices in order to consult with each other and to share information, including pictures, videos of themselves describing instruments and procedures, YouTube videos, and other pertinent information [42]. Using the app, information was easy to access and readily available and was positively reviewed by the operating room nurses [42].

Nurses used their smartphones for information seeking for both patient education purposes and for their own educational needs. Out of 22 studies, 4 (18%) reported that nurses used their smartphones for patient education [27,29,33,40]. Flynn et al [27] found that more than 75% of the nurses preferred the use of their smartphones to access information for patient education, and this was more commonly noted among nurses between the ages of 18 and 30 years compared to nurses older than 50 years of age. Furthermore, Flynn et al [27] discussed how nurses used their own smartphones to respond to patients’ and families’ requests for information on a variety of topics, such as medical procedures, medications, contact information for providers, and directions to local venues.

Personal smartphone use provided nurses with ease of access to information. For example, when asked by a patient about lab values, the nurse used their phone to search Google for the answer in the moment [40]. McBride and Levasseur [33] reported that nurses also used their personal smartphones to access patient handouts and teaching materials. Nurses accessed continuing education and professional development opportunities through their smartphones [30,33], and Mobasheri et al [34] reported that nurses used medical apps to enhance their clinical knowledge and skills.

Other uses of smartphones by nurses included the use of online evidenced-based guidelines to support patient care [26,30], to access information related to patients’ prognoses [29], to support nurses’ understanding of disease pathophysiology and physiology [30], and to access clinical decision support tools [35]. Multiple smartphone apps that were reported being used by nurses for information seeking included WebMD [28,40], Medscape [23,28,40], Google [28,40,43], UpToDate [23], Infermera virtual [31], and 061 CatSalut Respon [31]. Garner et al [28] reported nurses’ use of PubMed, YouTube, Medical in Nursing and Oncology App, Google Scholar, Q Calc, Doc Plus, Praco, Radiopedia, and Wikipedia as apps and programs on their smartphones. Giles-Smith et al [43] identified Lexicomp, Medscape, and iTriage as additional health-related apps on nurses’ smartphones for information seeking and patient care purposes. Nurses, particularly those with less than 5 years of clinical experience, used e-books on their personal smartphones for information seeking [24].

#### Communication

Nurses used their personal smartphones to communicate with members of the health care team. Out of 22 studies, 11 (50%) found that nurses used their smartphones while at work to connect with health care team members by calling and/or text messaging for patient care purposes [24-28,30,33,34,37,39,40]. Nurses reported improved communication between team members, including physicians, and other allied health care providers and claimed that their personal device use improved efficiency in communication and facilitated immediate contact with colleagues [27,39,40]. Voice calling and text messaging with nurses and physicians was the most commonly cited reason for personal smartphone use at work [24].

Bautista and Lin [40] reported that nurses were able to contact doctors immediately using the Viber app and also received patient care orders via their smartphones. Nurses reported an enhanced efficiency in their workflow because they did not need to “track down” the attending physician; instead, they used their smartphones to contact them immediately [39]. Another efficiency-related technique was the development of a directory of personal contacts, whereby nurses saved the contact information (eg, phone numbers) of physicians, supervisors, and colleagues across diverse organizational departments to their smartphones so they were readily available when required [27]. Specific apps used for communication among the health care team included Viber, Facebook Messenger, iMessage, WhatsApp, and Line [25,28,37,39]. Group chats were also mentioned, but a particular app was not reported for group chat purposes [39].

Nurses also used their smartphones to communicate with patients [41]. Chiang and Wang [41] assessed nurses’ experiences using the Line app to communicate with home care patients and families. Home care patients or their family members would contact the nurse regarding their health care via the text option on the Line app [41]. Chiang and Wang [41] found that the picture and video function on the app was used as an assessment tool; home care patients would take pictures and videos of signs and symptoms related to their health condition and send them to the nurse using the app. Nurses found the Line app beneficial because of the asynchronous nature of communication patterns, which afforded them time to problem solve patient care issues, and the unlimited texting function increased opportunities for patient-provider communication [41]. However, nurses also reported a situation of being chronically “on call,” where patient text messages were perceived as intrusive outside of their working hours [41]. In another study, which investigated the use of WhatsApp, researchers found that nurses rarely communicated with patients, whereas in the same study, physicians were more likely to communicate with patients using WhatsApp [25].

#### Mobile Device Functions

Three smartphone functions were identified as useful: the photo and video function [24,27,34,39,40], the calculator [26,27,31,33-35,37,38], and the flashlight function [27,44]. Nurses used the photo and video function on their smartphones to support communication with their colleagues. For example, nurses took pictures of radiology images, patient wound sites, electrocardiograms, procedural equipment, skin test results, and biological samples, such as sputum [27,40], to facilitate communication with other care providers who requested greater details of the patient condition beyond verbal reports [27].

Flynn et al [27] reported that over 75% of the nurses in their study valued the calculator function on their smartphones. The calculator was used to compute nursing and medical formulas and to calculate dose and scale [26,31,37]. Other than reported use, no further details were provided regarding the flashlight function.

### Implications of Personal Smartphone Use

#### Smartphone Use for Personal Reasons

In 12 out of 22 (55%) studies, nurses used their smartphones within the workplace setting for nonwork-related reasons [23,25-28,30,32,33,36,37,39,44]. Nurses’ most commonly reported smartphone use included texting and messaging family and friends [25,27,28,30,32,33,36,37,39,44]. The second-most common personal smartphone use was to check or post information on their personal social media sites [23,26,28,30,32,33,36,37,44]. Other common uses were for telephone calling or checking for missed calls [27,28,30,36,44] and for entertainment such as playing games or shopping [26,32,33,44]. Personal use was reported outside of scheduled breaks [32,33]. Uses that were only mentioned once throughout the literature included internet access and surfing [30], catharsis and conveying grievances about their work with other nurses [39], the use of the global positioning system to verify their children’s location [27], and for personal safety (ie, walking to vehicles at night) [27].

#### Distraction

Distraction from patient care was identified by nurses as a concern regarding smartphone use within the workplace [23,25-27,30,33,37,40,43]. Nurses self-reported instances of their own distraction from patient care activities resulting from the use of their smartphones but also reported observed instances of inattentiveness among their nursing colleagues [23,26,30,33,37]. Pucciarelli et al [37] reported that 42% of nurses felt that smartphones were a distraction. For example, a nurse was distracted from inserting an intravenous line by the ringing sound of an incoming call on their smartphones [40], and 12.5% of nurses had witnessed a coworker make a medical error that they attributed to perceived distraction related to their smartphone use [33]. Alameddine et al [23] similarly found that 55% of nurses had witnessed distraction they attributed to smartphone use. Di Muzio et al [26] found that almost 62% of nurses thought that the use of personal devices could increase the risk of errors.

#### Patient Perception

Similarly, nurses expressed their lack of knowledge about patients’ perceptions of their smartphone use and the potential for patient complaints [27,28,35,40,43]. Nurses were concerned about patients’ possible negative feelings and thoughts about mobile devices being used at the beside [23,43].

#### Privacy and Confidentiality

Nurses identified privacy and confidentiality of patient health information as a concern associated with the use of their personal devices within the workplace [25,28,34,40,41]. Mobasheri et al [34] found that 3.6% of nurses believed there was patient-related clinical information retained on their personal smartphones. Similarly, home care nurses discussed their concerns regarding their use of the Line app, reporting that “others could easily see patients’ recent whereabouts or sensitive personal information on the software platform” [41].

#### Lack of Organizational Support and Policy Confusion

Lack of organizational support and policy confusion was expressed by nurses [30,40,41,43]. Despite organizational policy that prohibited personal smartphone use within their clinical setting, most nurses considered their smartphones as helpful technology in their workplace. Some nurses were aware of policies restricting personal smartphone use, while others stated they did not know what the existing policies for personal smartphone use entailed. Hranchook et al [30] reported that 47.2% of the nurse participants in their study knew that their organization had a personal device use policy, 22.6% said their institution did not, while 30.2% were unsure. When asked if medical administrators should develop a code of conduct for smartphone use to minimize unnecessary distraction, 44% of participants agreed, while 51% disagreed [23]. Nurses also reported barriers to Wi-Fi and internet access issues within their organizations that impeded their access to online resources [28,29,38,43].

## Discussion

### Principal Findings

A scoping review of 22 studies was conducted to examine the current breadth and range of research on nurses’ use of personal smartphones in the workplace; to our knowledge, this is the first review of its kind. Based on the studies included in this review, nurses have reportedly been using their personal smartphones within their workplace for personal and patient care purposes since 2013.

From the existing evidence, we know that nurses used their personal smartphone devices to gather patient care information and to communicate within the health care team. In support of evidence-informed patient care, nurses used online apps and programs to locate information relating to patient-prescribed medications, clinical procedures, diagnoses, laboratory tests, and more. The information-seeking and consultation behaviors align with evidence-informed nursing practice [18,45]. Nurses require access to specialized knowledge and comprehensive clinical information to inform their clinical decision making. However, the significant demands on nurses’ time impedes their ability to engage with research and health care resources required for evidence-informed practice [46]. The use of their personal smartphone devices provided nurses with rapid and easy access to online health information. It is possible that nurses are using their personal smartphones as a way to efficiently access needed resources and support their patient care information needs.

Despite the high level of smartphone and app use, there was minimal reporting on nurses’ attention to the quality and accuracy of the information garnered from the apps used. The commercial profitability of health-related apps tend to take priority over ensuring that apps are critically appraised for information accuracy [47]. However, when asked, nurses expressed that they want health apps to be certified by a health or professional institution, exemplifying their concern about information quality [31]. Nurses who conducted a risk assessment on the apps that they used assessed the trustworthiness of the source, sought guidance from professional bodies on technology and app use, and were vigilant about patient information privacy [35].

Similarly, nurses may be resorting to their own personal digital devices to compensate for the lack of support provided by their organizations [42]. As reported elsewhere, nurses who have access to resources from a medical library, have internet access at work, and have the opportunity to work with computer technologies are more likely to engage in evidence-informed nursing practice [48]. Organizations that strive to provide the highest-quality patient care are also accountable to providing the resources that make this achievable. The presupposition of health information technology within the clinical setting highlights the need for organizations to provide continuing education to all health care providers regarding risk assessments of health information technologies (eg, devices and apps) for responsible use and to support evidence-informed practices.

Effective communication among the health care team is essential for quality patient care and effective teamwork [49]. In fact, the most common reason for errors resulting in patient harm result from communication failures [50]. In our review, nurses used text messaging, telephone, and picture and video functions on their smartphones to communicate with other members of the health care team. Furthermore, nurses preferred the use of their own smartphones for efficient and immediate communication among health care team members. Medical students have also reported using personal smartphones as an efficient means of communication and for coordination of the clinical team [51]. It may be that organizationally based communication systems do not provide satisfactory options to meet the precipitous communication needs of health care teams working within a digital health context [52]. Similarly, nurses’ use of their personal devices to communicate among members of the health care team may demonstrate a technological “work-around” meant to reconcile health system demands for cost-efficiency with efforts to provide quality patient care [53].

Despite nurses’ desires to use their personal smartphones in the workplace, they were also mindful of concerns that included personal and/or colleague distraction, and they acknowledged challenges to information privacy and information security related to possible retention of patient data on their smartphones. Nurses’ use of their personal devices to share practice-related information among health care teams may characterize their “work-around” of organizational health information systems that have not effectively considered nurses’ workflows, their need for accessible and up-to-date health care information, nor communication practices among multidisciplinary health care teams [52,54]. Nurses are not the only health professionals experiencing these tensions. Physicians report that barriers to using their smartphones in practice include organizational policies against smartphone use and concerns about using them during patient consultations [9]. The tension between quality health practices and pressure to find system efficiencies may have also contributed to nurses’ use of their personal smartphones within the workplace [54]. The reported lack of awareness of organizational policies related to personal smartphone use and nurses’ lack of knowledge regarding patient perception of their smartphone use for patient care constitute two important areas for future research and health professional education [55].

### Implications

#### Implications for Research

This scoping review highlights significant gaps in research regarding nurses’ use of personal smartphones in the workplace. To date, research has focused on acute care nurses, with little investigation of nurses’ device use within other practice settings. Research is needed to understand personal smartphone use across a more diverse range of health care settings, such as home care, long-term care, and public health. A greater understanding of other health care settings, such as long-term care, will provide invaluable information, especially due to the aging population in Canada and the growing demand on care providers for the elderly. COVID-19 exposed profound gaps in long-term care in Canada, including access to practice standards and education. The importance of public health care workers was also emphasized during the COVID-19 pandemic. Research can help to inform how smartphones can be leveraged to support nurses, especially during resource and staffing shortages. This will also help to generate a greater understanding of the online resources (eg, online information sites and apps) that support nurses’ practices. Research is also needed to inform smartphone practice strategies and policies that support efficient team communication and that are accountable to information, privacy, and security concerns.

#### Implications for Practice

Nurses are using their personal devices to access clinical information and to contribute to clinical decision making. If apps are being used and patient information is entered, it is important to know where the information is being stored, who has access to it, and who owns the data. Nurses’ use of their personal digital devices for sharing patient-related information within the health care team carries a potential risk for inappropriate disclosure of personal health information. While unapologetic about using their personal devices, nurses instead advocated for information-secure messaging apps that would ensure the safety and security of people’s personal health information [34].

#### Implications for Policy

Evidence that over 75% of nurses and physicians within acute care settings use their personal smartphones to support their patient care practices is indicative of a clear gap between policy and practice [27,38]. If nurses, as well as other health care professionals, use their smartphones as an efficient way to retrieve and share information, it would be incumbent on organizations to reassess their policies regarding personal phone use and to work with technology developers and care providers to nurture their innovative thinking and insight into effective, responsive, and responsible technology-enabled solutions.

### Limitations

There were several limitations when completing this scoping review. As 16 of the 22 studies were quantitative and used cross-sectional surveys to gather data, there were limited descriptions and examples available to provide an in-depth understanding of smartphone and mobile phone use. In addition, due to the lack of diversity in the target population, there is limited evidence across the nursing continuum. Lastly, studies included were only in English; studies in other languages were not included in these findings.

### Conclusions

This scoping review provides insight into nurses’ use of their personal smartphones within the workplace setting. The review highlights knowledge gaps regarding nurses’ personal device use and the safety and privacy of personal health information, patient care outcomes, communication practices among health care teams, and insight into app and information accuracy and reliability, thus creating significant opportunities for future research regarding nurses’ use of personal digital technology in the workplace. Organizational policies that limit or prohibit smartphone use may be shortsighted; insightful leadership would leverage the knowledge of nurses with other health care providers to collaboratively develop strategies that enable efficient, respectful, and ethical use of communication technology for effective patient care practices [56].

## Appendix

Multimedia Appendix 1 Full syntax of search terms for the literature search process.

## Abbreviations

PRISMA-ScR: Preferred Reporting Items for Systematic Reviews and Meta-Analyses extension for Scoping Reviews
PRN: *pro re nata*
