# Supplementary material for: Nurses’ Use of Personal Smartphone Technology in the Workplace: Scoping Review
Source: JMIR Mhealth Uhealth. 2020 Nov 26;8(11):e18774. doi: 10.2196/18774 (PMC7728531; doi:10.2196/18774)
Supplement: Multimedia Appendix 1 [file mhealth_v8i11e18774_app1.pdf]

Supplementary Material 1: Search Terms

Search terms:

Nurse terms: (nurse OR nursing OR nurses)

Personal digital technology terms: (personal digital technology OR smartphone OR mobile phone OR cellular phone OR cell phone).

Limits:

English only
2010-present
